# Plasma Proteome-Driven Liquid Biopsy for Individualized Monitoring and Risk Stratification of Immune-Related Adverse Events in Checkpoint Immunotherapy

**DOI:** 10.1016/j.mcpro.2025.101488

**Published:** 2025-12-13

**Authors:** Dongxue Yan, Jingjing Xu, Dawei Wang, Qian Xing, Xinrong He, Donghao Wang, Biao zhu, Kaijiang Yu, Meng Zhou, Changsong Wang

**Affiliations:** 1 Department of Critical Care Medicine, The First Affiliated Hospital of Harbin Medical University, Harbin Medical University, Harbin, P. R. China; 2 School of Biomedical Engineering, Wenzhou Medical University, Wenzhou, China; 3 Department of Critical Care Medicine, Harbin Medical University Cancer Hospital, Harbin Medical University, Harbin, Heilongjiang, P. R. China; 4 Heilongjiang Provincial Key Laboratory of Critical Care Medicine, Harbin Medical University, Harbin, P. R. China; 5 Department of ICU, Fudan University Shanghai Cancer Center, Shanghai, P. R. China; 6 Department of Anathesia, Critical Care and Pain, Fudan University Shanghai Cancer Center, Shanghai, P. R. China; 7 Department of Intensive Care Medicine, Sun Yat-Sen University Cancer Center, Guangzhou, P. R. China; 8 Department of Intensive Care Medicine, Tianjin Medical University Cancer Hospital, Tianjin, P. R. China

## Abstract

Immune checkpoint inhibitors (ICIs) have revolutionized cancer therapy, however, their use is limited by heterogeneous and unpredictable immune-related adverse events (irAEs), which can progress to life-threatening conditions requiring intensive care unit (ICU) admission. Reliable biomarkers for predicting and stratifying ICU-level irAEs are urgently needed to improve immunotherapy safety and critical care management. Here, we performed comprehensive mass spectrometry-based proteomic profiling to identify plasma biomarkers for the prediction and monitoring of irAEs in 65 patients receiving ICI treatment. Our analysis identified 217 differentially abundant proteins and four co-expression modules related to humoral (antibody-mediated) and cellular (T cell-mediated) immunity spanning mild to severe irAEs. Through feature selection and cross-validation with proteomics and ELISA data, we identified two key proteins, *IL1RL1* and *FABP3*, as potential biomarkers for irAE risk. In addition, we developed a plasma proteomic machine learning model (ProIRAE) that demonstrated high and robust predictive performance with area under the receiver-operating characteristic curve (AUROC) values of 0.929 and 0.766 for identifying patients at risk of developing irAEs, and AUROC values of 0.978 and 1.000 for predicting severe irAEs in the discovery and independent validation cohorts, respectively. Collectively, our study provides a valuable plasma proteomic atlas of ICI-related irAEs. The ProIRAE model offers a non-invasive tool for the detection and severity stratification of irAEs, with a great potential to improve precision monitoring and management of immunotherapy complications in critical care settings.

## Highlights

• Shift from humoral to cellular immunity as irAEs increase in severity.
• Persistent inflammation/chemotaxis up (M1) and complement/coagulation down (M4).
• ProIRAE, a plasma dual-protein model (***IL1RL1*** and ***FABP3***) for irAE risk monitoring.

## Graphical Abstract

**Figure:**
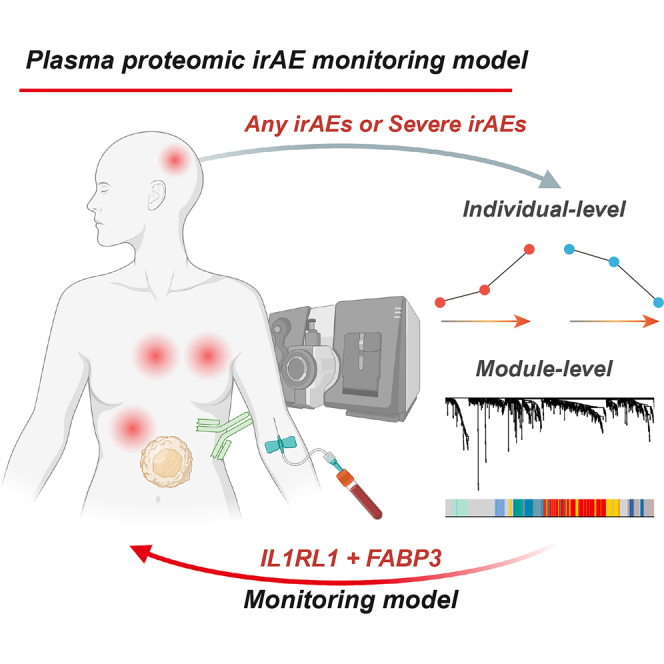

## In Brief

ProIRAE, a plasma dual-protein model (IL1RL1 and FABP3), improves risk stratification for immune-related adverse events in checkpoint immunotherapy.

Immune checkpoint inhibitors (ICIs) have revolutionized cancer treatment by harnessing the immune system to target tumor cells and have become a cornerstone of modern cancer immunotherapy (1). Both the therapeutic mechanism of ICIs and the development of its high burden of toxicity, known as immune-related adverse events (irAEs), originate from an over-activated immune response and lead to immune-mediated damage to multiple organs (2, 3). Consequently, achieving a balance between treatment efficacy and substantial therapy-related toxicity remains crucial (3, 4, 5). The onset of irAEs is highly variable and often unpredictable, occurring anywhere from a few weeks to several months after the initiation of immunotherapy (6, 7), with manifestations ranging from mild, transient symptoms to severe, life-threatening conditions requiring intensive care unit (ICU) admission (2, 8, 9).

Some studies on toxicity and efficacy suggest that the occurrence of irAEs may be associated with improved treatment outcomes, generally following a trend where a larger number of irAEs correlate with better efficacy (10, 11, 12). However, this positive correlation likely may hold within an acceptable toxicity range (13, 14, 15), especially for low-grade toxicities, like dermatological and gastrointestinal events. In contrast, high-grade irAEs can still lead to treatment discontinuation, disability, or even mortality (8, 16). The unpredictable nature and variable severity of irAEs highlight the urgent need for reliable biomarkers to enable non-invasive monitoring and effective management, particularly for ICU-level complications (17). Recent studies have shown the growing use of blood samples as diagnostic tools for irAEs in clinical settings owing to their non-invasiveness, ease of collection and preservation (18, 19, 20). Proteins, as products of gene expression, have proven valuable in identifying preexisting disease-specific autoantibodies and non-disease-specific cytokine markers associated with irAE development (3, 18, 21, 22). For example, serological analyses using recombinant cDNA expression have shown that anti-CD74 antibodies serve as markers of ICI-induced pneumonitis, whereas anti-GNAL and anti-ITM2B antibodies are associated with hypophysitis (23). In addition, studies of selected cytokines have consistently shown increased cytokine responses with irAE development (24, 25), and a combined cytokine score involving 11 cytokines has been proposed as a potential predictor of severe irAEs (26). Furthermore, some studies have expanded their focus beyond limited cytokines to a broader inflammation panel using Olink proteomic analysis, given the widespread involvement of proteins in irAEs (27), although protein coverage is still limited. Despite these advances, irAEs remain poorly characterized, and preclinical models and sufficient clinical data are lacking. Therefore, there is a critical need for specific and sensitive biomarkers to predict, monitor, and stratify irAEs, particularly those progressing to ICU-level severity.

In this study, we conducted a comprehensive analysis of plasma proteomic alterations associated with varying severities of irAEs across a pan-cancer cohort of patients receiving anti-PD-1/PD-L1 immunotherapy as (neo)adjuvant therapy in combination with chemotherapy. We developed a plasma proteomic irAE risk prediction model (ProIRAE) for non-invasively monitoring the transition from a non-irAE state to an active irAE and risk stratification of irAEs in checkpoint immunotherapy. The performance of the ProIRAE model was further validated through enzyme-linked immunosorbent assay (ELISA) and proteomic profiling in an independent validation cohort. Our findings provide a valuable resource for improving the management of irAEs and guiding more personalized, effective, and safe immunotherapy regimens for patients with cancer, particularly in critical care settings.

## Experimental Procedures

### Experimental Design

This retrospective study employed a mass spectrometry-based discovery-validation cohort design and was conducted at Harbin Medical University Cancer Hospital from August 2020 to April 2024. A total of 65 patients receiving anti-PD-1/PD-L1 blockade therapies as (neo)adjuvant therapy in combination with chemotherapy were enrolled and categorized into two cohorts: a discovery cohort comprising 47 participants recruited between August 2020 and February 2023, and a validation cohort consisting of 18 participants recruited between April 2023 and April 2024 to assess generalizability. Key proteins identified in the discovery set were further validated using ELISA to confirm their stability. Ethical approval was obtained from the Ethics Committee of Harbin Medical University Cancer Hospital (approval number: kyk2020003), and the study adhered to the guidelines of the Declaration of Helsinki. Informed consent was obtained from all participants before enrollment.

### Participant Cohorts and Sample Collection

Eligible participants were aged between 30 and 75 years and had received anti-PD-1/PD-L1 therapies for at least 1 week. Participants with a history of immune system diseases (including HIV, systemic lupus erythematosus, hyperthyroidism, or Cushing's syndrome) or those who had undergone organ transplantation within the past 6 months were excluded from the study. Clinical information collected from the participants included sex, age, body mass index (BMI), treatment cycle of therapy, cancer type, and stage.

All irAEs were assessed based on the National Comprehensive Cancer Network (NCCN) guidelines (version 1.2020). The severity of irAEs was assessed based on the U.S. Department of Health and Human Services' Common Terminology Criteria for Adverse Events (CTCAE, version 5.0). These irAEs affected various organ systems, including but not limited to the skin, liver, lungs, kidneys, nervous system, and cardiovascular system, as detailed in Supplemental Table S1. Based on the severity of irAEs, participants were categorized into three groups: (1) Control group: participants who did not experience any irAEs, at least 14 days after the end of the previous immunotherapy cycle and before the initiation of the next cycle; (2) Mild group: participants with all irAEs classified as grade 1 or 2; and (3) Severe group: participants with any grade 3 or higher irAEs admitted to the ICU. Plasma samples were obtained from all the participants. For the Mild and Severe groups, plasma samples were collected on the first day of irAE diagnosis.

### Plasma Protein Extraction and Trypsin Digestion

Blood samples were collected in EDTA tubes (BD Vacutainer, Beckton Dickinson) and centrifuged at 1000*g* for 10 min at 4 °C within 2 h of sampling. The plasma was further centrifuged at 12,000*g* at 4 °C for 10 min to remove cellular debris. The supernatant was transferred to a new centrifuge tube. High-abundance proteins were depleted using the Pierce Top 14 Abundant Protein Depletion Spin Column Kit (Thermo Fisher). Protein concentration was determined using a BCA assay kit according to the manufacturer’s instructions.

Equal amounts of protein from each sample were used for digestion. The sample volumes were adjusted with lysis buffer to ensure consistency. The protein solution was reduced with 5 mM dithiothreitol at 56 °C for 30 min, followed by alkylation with 11 mM iodoacetamide at room temperature in the dark for 15 min. The alkylated samples were transferred to ultrafiltration units and centrifuged at 12,000*g* for 20 min at room temperature. The samples were washed three times with 8 M urea, followed by three washes with buffer solution. Trypsin was added at a 1:50 enzyme-to-protein mass ratio, and digestion was performed overnight. The resulting peptides were collected by centrifugation at 12,000*g* for 10 min at room temperature, followed by additional washing with double-distilled water. The combined peptide solutions were reserved for liquid chromatography-tandem mass spectrometry (LC-MS/MS) analysis.

### LC-MS/MS Analyses

The peptides were dissolved in solvent A (0.1% formic acid and 2% acetonitrile in water) and separated using a Vanquish UHPLC system. Peptides were separated using a gradient from 7% to 20% solvent B (0.1% formic acid in 90% acetonitrile) for 16 min, 20% to 32% for 8 min, and then increased to 80% for 6 min. The flow rate was maintained at 500 nl/min. After separation, the peptides were ionized using a nanospray ionization (NSI) source and analyzed with an Orbitrap Exploris 480 mass spectrometer. The ion source voltage was set to 2.3 kV, and the FAIMS compensation voltages were set to −45V and −70V. Both precursor and fragment ions were detected and analyzed with high resolution using Orbitrap.

Data acquisition was performed in data-independent acquisition (DIA) mode. The full MS1 scan was set to a resolution of 30,000 across a range of 390 to 810 m/z. Subsequently, scans were divided into 23 consecutive m/z isolation windows, with an average width of approximately 17 m/z. The precursor ions within each window were fragmented in the higher-energy collisional dissociation (HCD) collision cell with normalized collision energies of 25%, 30%, and 35%, followed by sequential MS2 analysis. The resolution of MS2 was set to 30,000, with a fixed starting point of 200 m/z. To maximize mass spectrometry utilization efficiency, the automatic gain control (AGC) target was set to 3e6 with the maximum injection time set to “auto”.

### Peptide Identification and Protein Quantification

The resulting DIA data were searched against the UniProt human protein database (updated on 2023–01–03, 20,389 entries) using Spectronaut (v17) with the Pulsar search engine with default settings. In the Spectronaut analysis, the mass tolerances for both MS1 and MS2 were set to ±40 ppm. A reverse decoy database was included in the search to calculate the false discovery rate (FDR) and to account for random matches. Trypsin/P was specified as the cleavage enzyme, allowing up to two missed cleavages. Cysteine carbamidomethylation was specified as a fixed modification, whereas acetylation at protein N-terminals and oxidation at methionine residues were specified as variable modifications. The FDR thresholds were set to 1% for peptide-to-spectrum match, peptide, and protein identification and quantification.

### Proteomics Data Quality Control and Preprocessing

Protein abundance values less than 10 were marked as missing values (NA). Proteins identified in less than 50% of the samples were excluded from further analysis, and the remaining proteins with log2-transformed values were imputed using the k-nearest neighbor (k-NN) method with a parameter setting of k = 10 using the R package statistical and bioinformatic analyses. Median centering were used for batch effect before model construction (28).

### Differential Protein Analysis

Differential protein abundance analysis was performed to identify proteins with significant differences between the Control, Mild and Severe irAE groups. Proteins with consistently upregulated or downregulated monotonic trends were identified using the Kruskal-Wallis tests and two-sided Jonckheere-Terpstra tests. The direction of these trends was determined based on the median protein intensity across groups. The two-sided Wilcoxon rank-sum test was used to compare protein abundance between the Control vs. Mild, Mild vs. Severe, and Control vs. Severe groups as well as between irAEs vs. no irAEs and severe irAEs vs. no severe irAEs for proteins with strong trend changes. Proteins with an FDR < 0.05 were considered significantly different in abundance.

### Functional Annotation and Enrichment Analysis

Secretory and tissue-specific proteins were annotated according to the Human Protein Atlas (HPA). Differentially expressed proteins were subjected to Gene Ontology (GO), KEGG pathway, and Reactome enrichment analyses using the R packages 'clusterProfiler' and 'ReactomePA', with an FDR of **<** 0.05.

### Weighted Correlation Network Analysis (WGCNA)

WGCNA was performed to identify protein co-expression modules in an unsupervised clustering manner using the blockwiseModules function in the R package “WGCNA” with the following settings: soft power = 6, bidweight mid-correlations (bicor) for correlation calculation, deepSplit = 4, a minimum module size of 14, merge cut height of 0.07, mean TOM denominator, a signed network, a minimum kME of 0.4 for protein retention within a module, and a reassignment threshold of *p* < 0.05.

We used the WGCNA::mergeCloseModules(XX) function to reduce the number of modules with high similarity in module eigenproteins (representative abundance values for each module). Proteins were reassigned based on Spearman’s correlations, with a maximum dissimilarity threshold of 0.25. Module preservation was assessed across different levels of proteome missingness using the WGCNA::modulePreservation(XXX) function, with unassigned proteins labeled as M0 and colored gray.

### Enzyme-Linked Immunosorbent Assay (ELISA)

Plasma protein levels were quantified using commercially available ELISA kits according to the manufacturer’s instructions. The specific kits used in this study were as follows: *IL1RL1* (CSB-E13789h), *ADM* (CSB-E09146h), *FABP3* (CSB-E09185h), *TNC* (CSB-E13125h), *TNFIP6* (CSB-EL023959HU), *VSIG4* (CSB-EL025928HU), *PLIN3* (MM-63554H2), and *VWCE* (MM-51800H2). All experimental procedures adhered to the protocols provided by the kit manufacturers.

### Machine Learning-Based Protein Model for irAE Risk Prediction

To ensure the clinical relevance and robustness of the protein variables used for model construction, recursive feature elimination based on random forest (RFE-RF) was employed as the primary feature selection method among the ELISA-validated proteins. Eight distinct machine learning algorithms were then constructed and evaluated using five-fold cross-validation to prevent overfitting, including: Naïve Bayes, ordinal logistic regression, multilayer perceptron (MLP), random forest, support vector machine (SVM), elastic net, XGBoost, and decision tree. Hyperparameter optimization was systematically performed via grid search. Specifically, for the ordinal logistic regression model, the optimal “link” function and “family” parameters were determined using the R package ordinalNet, while hyperparameter tuning for the remaining seven algorithms was conducted using the caret package. The final model, designated as ProIRAE, was selected based on a performance heatmap derived from five-fold cross-validation, retrained on the entire discovery cohort, and subsequently applied to the independent validation cohort. The optimal cut-off value was determined by maximizing Youden's Index from the ROC curve analysis in the discovery cohort.

### Statistical Analyses

All statistical analyses were performed using R (v4.2.0) and associated packages. Group comparisons for continuous variables were conducted using the two-sided Wilcoxon rank-sum test or Kruskal-Wallis test, where appropriate. Fisher's exact test was used to analyze categorical variables. Trend analysis across ordered severity groups was performed using the Jonckheere-Terpstra test. Model performance was assessed using area under the receiver–operating characteristic curve (AUROC) and area under the precision-recall curve (AUPR), with 95% confidence intervals calculated via 1000 bootstrap iterations. For classification performance evaluation, confusion matrices were constructed to calculate key metrics, including sensitivity and specificity.

## Results

### Study Design and Clinical Characteristics of the irAE Cohort

**Fig. 1:**
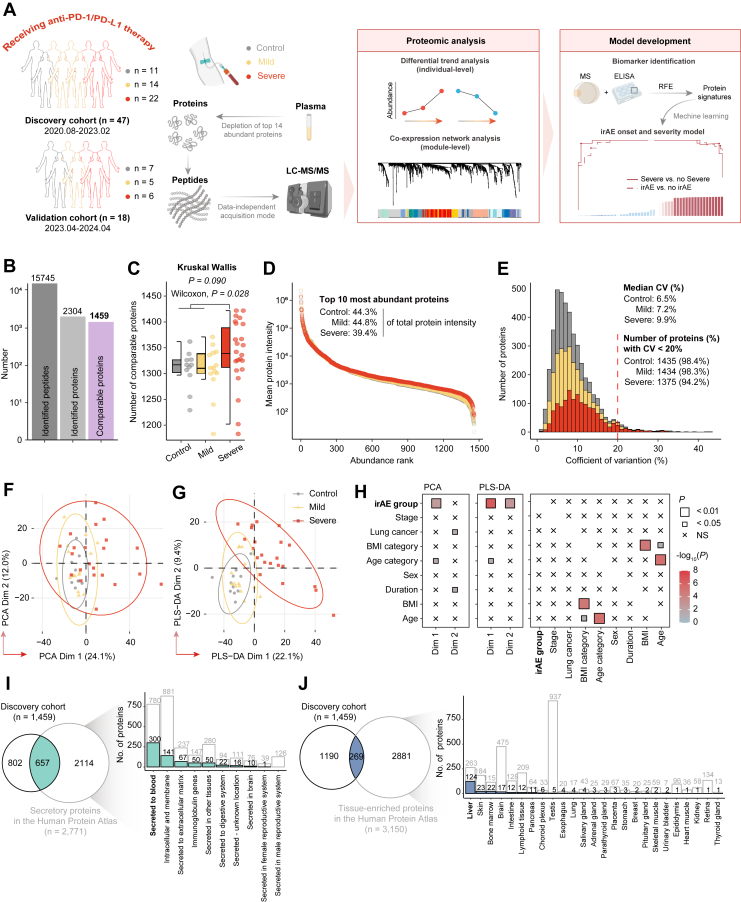
**Overview of the plasma proteomic analysis of irAE cohorts.***A*, overview of the plasma proteomic workflow, including cohort construction [plasma discovery cohort (Control: N = 11; Mild irAEs: N = 14 and Severe irAEs, N = 22) and plasma validation cohort (Control: N = 7; Mild irAEs: N = 5 and Severe irAEs, N = 6)], proteomic profiling (data-independent acquisition mass spectrometry and enzyme-linked immunosorbent assay), and data analysis. *B*, number of peptides and proteins identified during the quality control process. *C*, number of comparable proteins across different irAE groups. *D*, the dynamic range of the protein identified in different irAE groups according to the descending sort of protein abundance. *E*, distribution of coefficient of variations for protein abundance across irAE groups. *F*, Principal component analysis (PCA) of all comparable proteins measured in plasma samples. *G*, partial least squares discriminant analysis (PLS-DA) of all comparable proteins measured in plasma samples. *H*, correlation between clinical features and proteomic profiling of irAE severity, evaluated with PCA/PLS-DA scores. Statistical significance assessed using Pearson correlation coefficients for continuous variables, Fisher's exact test for categorical variables, and two-sided Wilcoxon or Kruskal-Wallis tests for continuous *versus* categorical variables. *I* and *J*, Secretory and tissue-specific proteins identified in samples annotated with the HPA.

To investigate plasma proteomic patterns and identify potential noninvasive biomarkers for ICI-associated irAEs, we conducted a comprehensive proteomic analysis of plasma samples. A total of 65 participants receiving anti-PD-1/PD-L1 therapies as (neo)adjuvant therapy in combination with chemotherapy were enrolled in this study and stratified into three groups based on irAE severity: Control (no irAEs), Mild (all irAEs grade < 3), and Severe (any irAEs grade ≥ 3). Participants were further divided into a discovery cohort of 47 participants (11 Control, 14 Mild, and 22 Severe) and a validation cohort of 18 participants (7 Control, 5 Mild, and 6 Severe), based on the time of recruitment (Fig. 1*A*). The discovery cohort was used for proteomic analysis, biomarker identification, and model construction, while the validation cohort was used to assess the generalizability and stability of the model.

The characteristics of participants in the irAE cohort are summarized in Table 1. The mean age ranged from 56.6 to 65.4 years, and the mean body mass index (BMI) ranged from 20.9 to 25.7. As all irAEs in this study resulted from the disruption of immune signaling pathways targeted by anti-PD-1/PD-L1 therapies, we used a pan-cancer approach. The most common cancer types among the participants were lung cancer and liver cancer, along with 13 other less common cancers.

**Table 1:** Baseline participant characteristic of irAE cohorts

Characteristics	Discovery cohort			Validation cohort		
	Control (N = 11)	Mild^a^ (N = 14)	Severe^a^ (N = 22)	Control (N = 7)	Mild^a^ (N = 5)	Severe^a^ (N = 6)
Age, years (mean ± sd)	62.5 ± 10.8	56.6 ± 11.9	63.6 ± 8.98	65.4 ± 6.27	61.8 ± 8.07	60.7 ± 8.45
Sex, n (%)						
Female	2 (18.2%)	2 (14.3%)	4 (18.2%)	5 (71.4%)	1 (20.0%)	2 (33.3%)
Male	9 (81.8%)	12 (85.7%)	18 (81.8%)	2 (28.6%)	4 (80.0%)	4 (66.7%)
BMI, kg/m^2^ (mean ± sd)	20.9 ± 2.86	24.4 ± 3.56	23.3 ± 4.14	21.7 ± 1.47	25.7 ± 3.60	21.9 ± 3.12
Treatment cycle (median [min, max])	3.00 [1.00, 10.0]	2.00 [1.00, 10.0]	1.50 [1.00, 5.00]	3.00 [1.00, 8.00]	6.00 [1.00, 22.0]	4.00 [1.00, 10.0]
Target, n (%)						
PD-1						
Nivolumab	1 (9.1%)	0 (0%)	0 (0%)	0 (0%)	1 (20.0%)	0 (0%)
Camrelizumab	4 (36.4%)	4 (28.6%)	14 (63.6%)	1 (14.3%)	2 (40.0%)	3 (50.0%)
Pembrolizumab	1 (9.1%)	3 (21.4%)	1 (4.5%)	0 (0%)	0 (0%)	0 (0%)
Sintilimab	4 (36.4%)	3 (21.4%)	4 (18.2%)	4 (57.1%)	0 (0%)	2 (33.3%)
Tislelizumab	1 (9.1%)	1 (7.1%)	1 (4.5%)	0 (0%)	2 (40.0%)	1 (16.7%)
Toripalimab	0 (0%)	0 (0%)	1 (4.5%)	0 (0%)	0 (0%)	0 (0%)
PD-L1						
Atezolizumab	0 (0%)	2 (14.3%)	1 (4.5%)	0 (0%)	0 (0%)	0 (0%)
Durvalumab	0 (0%)	1 (7.1%)	0 (0%)	1 (14.3%)	0 (0%)	0 (0%)
Envafolimab	0 (0%)	0 (0%)	0 (0%)	1 (14.3%)	0 (0%)	0 (0%)
Cancer type, n (%)						
Lung cancer	4 (36.4%)	3 (21.4%)	12 (54.5%)	1 (14.3%)	1 (20.0%)	2 (33.3%)
Liver cancer	4 (36.4%)	5 (35.7%)	0 (0%)	1 (14.3%)	2 (40.0%)	0 (0%)
Esophageal cancer	3 (27.3%)	2 (14.3%)	4 (18.2%)	1 (14.3%)	0 (0%)	1 (16.7%)
Colon cancer	0 (0%)	1 (7.1%)	0 (0%)	2 (28.6%)	2 (40.0%)	0 (0%)
Nasopharyngeal carcinoma	0 (0%)	0 (0%)	2 (9.1%)	0 (0%)	0 (0%)	0 (0%)
Gallbladder cancer	0 (0%)	0 (0%)	1 (4.5%)	0 (0%)	0 (0%)	0 (0%)
Lymphoma	0 (0%)	1 (7.1%)	0 (0%)	0 (0%)	0 (0%)	0 (0%)
Kidney cancer	0 (0%)	1 (7.1%)	1 (4.5%)	0 (0%)	0 (0%)	0 (0%)
Gastric cancer	0 (0%)	1 (7.1%)	0 (0%)	0 (0%)	0 (0%)	0 (0%)
Thymoma	0 (0%)	0 (0%)	1 (4.5%)	0 (0%)	0 (0%)	0 (0%)
Endometrial cancer	0 (0%)	0 (0%)	1 (4.5%)	0 (0%)	0 (0%)	0 (0%)
Bladder cancer	0 (0%)	0 (0%)	0 (0%)	0 (0%)	0 (0%)	1 (16.7%)
Cholangiocarcinoma	0 (0%)	0 (0%)	0 (0%)	1 (14.3%)	0 (0%)	0 (0%)
Peritoneal cancer	0 (0%)	0 (0%)	0 (0%)	1 (14.3%)	0 (0%)	0 (0%)
Cervical cancer	0 (0%)	0 (0%)	0 (0%)	0 (0%)	0 (0%)	2 (33.3%)
Stage, n (%)						
I	0 (0.0%)	0 (0.0%)	0 (0.0%)	1 (14.3%)	0 (0%)	0 (0%)
II	0 (0.0%)	0 (0.0%)	2 (9.1%)	1 (14.3%)	1 (20.0%)	3 (50.0%)
III	3 (27.3%)	2 (14.3%)	6 (27.3%)	0 (0%)	1 (20.0%)	1 (16.7%)
IV	8 (72.7%)	12 (85.7%)	14 (63.6%)	5 (71.4%)	3 (60.0%)	2 (33.3%)

^a^ All irAEs were classified according to the United States Health and Human Services Common Terminology Criteria for Adverse Events (CTCAE) v.5.0 with grade ≥3 considered to be severe.

### Plasma Proteomic Profiling of irAEs

A total of 15,745 peptides and 2304 proteins were identified in the 47 plasma samples from the discovery cohort using in-depth Blood DIA technology, with 1459 comparable proteins retained for further analysis after quality control (Fig. 1*B*). No significant differences in comparable proteomic coverage were observed between the Control group (median, 1316 proteins) and the Mild irAE group (median, 1311 proteins) (two-sided Wilcoxon rank-sum test, *p* = 0.956). However, significant differences were found between the Severe irAE group (median, 1339 proteins) and the other two groups (two-sided Wilcoxon rank-sum test, *p* = 0.027) (Fig. 1*C*). Protein intensities spanned over six orders of magnitude, with the 10 most abundant proteins accounting for 39.4% to 44.8% of the total protein abundance (Fig. 1*D*). Notably, the proteomic heterogeneity increased with irAE severity as evidenced by the heatmap for sample correlations and the increased median coefficient of variation (6.5% in Control, 7.2% in Mild irAEs, and 9.9% in Severe irAEs) (Fig 1*E* and Supplemental Fig. S1*A*).

Unsupervised principal component analysis (PCA) and supervised partial least squares discriminant analysis (PLS-DA) were used to compare and visualize proteomic profiles, revealing a potential discrimination between the three irAE groups, with the most pronounced separation observed for the Severe irAE group (Fig. 1, *F*–*H* and Supplemental Fig. S1*B*). Further annotation by the HPA identified 657 secretory proteins (45.0% of the analyzed proteins), of which 300 (45.7%) were actively secreted into circulation and 141 (21.5%) were membrane-spanning proteins (Fig. 1*I* and Supplemental Fig. S1*C*). Additionally, 269 tissue-specific proteins (18.4% of the analyzed proteins) were identified, the majority of which were liver-derived (124 proteins, 46.1%), indicating a substantial contribution of the liver to the plasma proteome (Fig. 1*J* and Supplemental Fig. S1*D*). Both secretory and tissue-specific protein profiles showed consistent patterns across the different irAE groups, with no significant correlations with other clinical characteristics. Taken together, these findings suggest that plasma proteomics can effectively reflect irAE severity.

### Identification of Potential Proteomic Biomarkers of irAEs

**Fig. 2:**
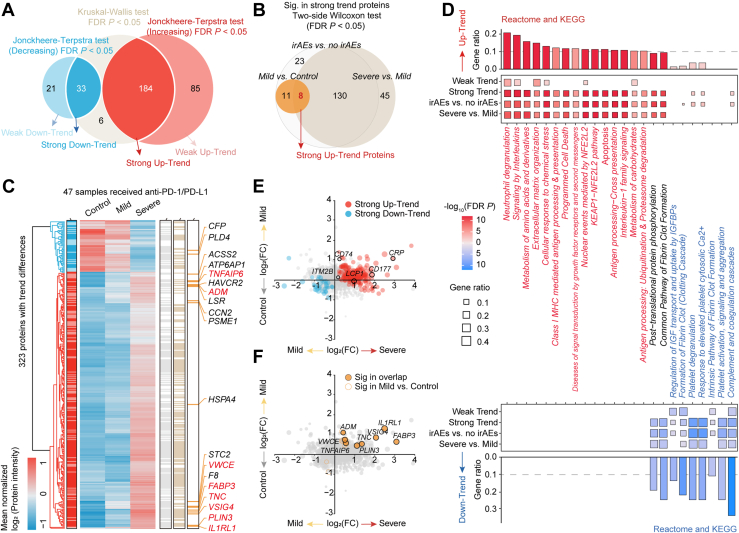
**Differential protein abundance analysis of plasma samples across irAE groups.***A*, Venn diagram showing proteins with differential abundance trends across irAE severity. *B*, Venn diagram showing proteins with strong differential trends across irAE groups. Proteins with significant differences between any two irAEs groups were marked in *red*. *C*, heatmap of proteins with differential abundance trends across irAE groups. *D*, Enriched KEGG and Reactome terms of proteins with differential abundance trends across irAE groups. Terms are marked as decreased (*blue*) or increased (*red*) (FDR *p* < 0.05 and gene ratio > 0.1). *E*, Scatter plots illustrating fold changes for proteins with strong or weak trend differences. *Black circles* denoted candidate irAEs biomarkers from previous literature, including *CRP*, *CD177*, *LCP1*, *CD74*, and *ITM2B*. Dots further from the origin indicated larger fold changes for Severe vs. Control. *F*, Scatter plots showing fold changes for shared proteins with significant differences between any two irAEs groups.

To further investigate the proteomic changes associated with irAE severity, we performed differential protein analysis and identified 217 proteins with significant alterations in abundance that correlated with monotonic progression of irAE severity. Of these, 184 proteins showed increased abundance, whereas 33 showed decreased abundance as irAE severity progressed (Fig. 2*A* and Supplemental Table S2). Additionally, 106 proteins demonstrated weaker shifts in abundance, with 85 showing increased levels, and 21 showing decreased levels. Proteins with pronounced strong trends were more significantly altered between the Mild and Severe irAE groups than between the Control and Mild groups, suggesting that proteomic changes become more pronounced as irAE severity increases (Fig. 2*B* and Supplemental Table S2).

Enrichment analysis revealed that dysregulated proteins with strong trends were more consistently altered across irAE severity groups compared to proteins with weaker trends, despite relatively uniform clustering in samples (Fig. 2, *C* and *D*, Supplemental Fig. S2, and Supplemental Table S3). Functional annotation using Reactome and KEGG pathway analyses revealed that proteins with strong trends were primarily involved in inflammatory and chemotactic responses (e.g., neutrophil degranulation, interleukin signaling, and interleukin-1 family signaling), metabolic and cellular responses (e.g., metabolism of amino acids and carbohydrates, extracellular matrix organization, and cellular response to chemical stress), and immune and antigen processing (e.g., class I MHC-mediated antigen processing and presentation and antigen processing: ubiquitination and proteasome degradation). In contrast, pathways related to complement and coagulation functions, as well as humoral immunity (e.g., antigen binding and immunoglobulin secretion), were consistently downregulated, indicating a shift from humoral (antibody-mediated) to cellular (T cell-mediated) immunity with increasing irAE severity (Fig. 2*D* and Supplemental Fig. S2).

Several previously identified irAE biomarkers were also differentially expressed in the plasma, including C-reactive protein (*CRP*) (associated with systemic inflammatory response; Kruskal-Wallis FDR *p* = 0.028), cluster of differentiation 177 (*CD177*) (involved in immune-mediated intestinal disease; Kruskal-Wallis FDR *p* = 0.009), and lymphocyte cytosolic protein 1 (*LCP1*) (Kruskal-Wallis FDR *p* = 0.009), despite their stable expression across the Control and Mild groups (Fig. 2*E*). By comparing the different irAE severity groups, we identified eight proteins that consistently escalated with increasing irAE severity (Fig. 2, *B* and *F*). These proteins highlight their potential as non-invasive biomarkers for predicting and monitoring irAE risk and progression in patients receiving anti-PD-1/PD-L1 therapies.

### Clinically Relevant Functional Protein Modules

**Fig. 3:**
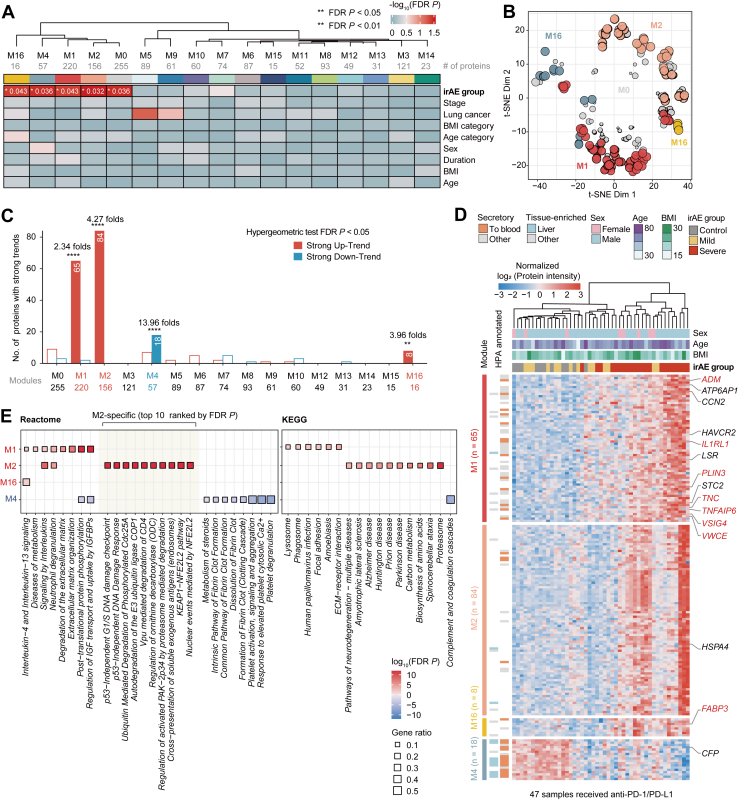
**Network analysis of plasma proteomics and clinically relevant functional protein modules.***A*, weighted gene co-expression network analysis (WGCNA) of plasma proteomics and correlation analysis of module eigenproteins (the first principal components of module protein expression) with irAE and clinical features. *B*, T-distributed stochastic neighbor embedding (t-SNE) analysis with top 25% proteins in kME value for each irAE-related network module. The size of dots referred to the value of module eigenproteins. *C*, number of proteins with strong differential trends co-detected in network modules. The two-sided hypergeometric tests (FDR *p* < 0.05) were performed, with the enrichment fold scored as the ratio of actual to expected hits. *D*, heatmap of hypergeometric test-identified proteins with strong differential trends, annotated with HPA, across irAE groups. *E*, KEGG and Reactome analysis for hypergeometric test-identified proteins in irAE-related network module. Terms are marked as decreased (*blue*) or increased (*red*) (FDR *p* < 0.05 and gene ratio >0.1).

To identify co-regulated proteins and their association with irAEs, we performed WGCNA on the proteomic data and identified 17 distinct protein modules, ranging in size from 255 proteins in M0 to 15 proteins in M16 (Supplemental Fig. S3, *A* and *B* and Supplemental Table S4). These modules remained highly stable across different data completeness thresholds (10% to 50%) (Supplemental Fig. S3*C*). Only five modules showed significant associations with irAE severity, indicating their involvement in irAE progression, independent of other clinical characteristics (Fig. 3*A*). Unsupervised hierarchical clustering further revealed that four key protein modules (M1, M2, M4, and M16) show clearly distinct clustering, suggesting potential association with the clinical manifestation of irAE (Fig. 3*B* and Supplemental Fig. S3*D*).

We then mapped 217 proteins with strong differential trends to these functional protein modules using hypergeometric tests (Fig. 3*C*). Three modules (M1, M2, and M16) were significantly enriched for proteins with upregulated trends, with M1 showing 2.34-fold enrichment, M2 showing 4.27-fold enrichment, and M16 showing 3.96-fold enrichment. In contrast, module M4 demonstrated a remarkable 13.96-fold enrichment of downregulated proteins. These detected proteins highlight the robust association between module composition and irAE progression, as the recalculated eigenproteins showed high correlations with the original module eigenproteins, ranging from 0.93 to 0.99 (Supplemental Fig. S3, *E* and *F*).

The key advantage of this network-based approach over analyzing differentially abundant proteins individually is its ability to uncover groups of co-regulated proteins (modules) with high topological overlap, which often correspond to coherent functional units. Functional enrichment analysis based on the KEGG and Reactome revealed that proteins within these modules not only marked differences in irAE severity but also correlated with specific biological processes and molecular pathways more clearly than the individual protein analysis suggested (Fig. 3, *D* and *E*, Supplemental Fig. S4 and Supplemental Table S5). We found that M1 was prominently involved in inflammatory and chemotactic pathways as well as in matrix organization, indicating increased T-cell activity. M2 was primarily associated with antigen–antibody interactions and disease-related pathways, whereas M4 featured a downward trend in complement and coagulation-related functions, referred to as decreased humoral activity (Fig. 3*D*, Supplemental Fig. S4). This distinction underscores the value of WGCNA in refining the functional interpretation of proteomic data beyond differential abundance alone. Among these modules, M1 and M4 exhibited consistent stability across samples from different irAE severity groups and were enriched in secretory proteins (Fig. 3*E* and Supplemental Fig. S5).

### Plasma Protein Risk Prediction Model for irAEs

**Fig. 4:**
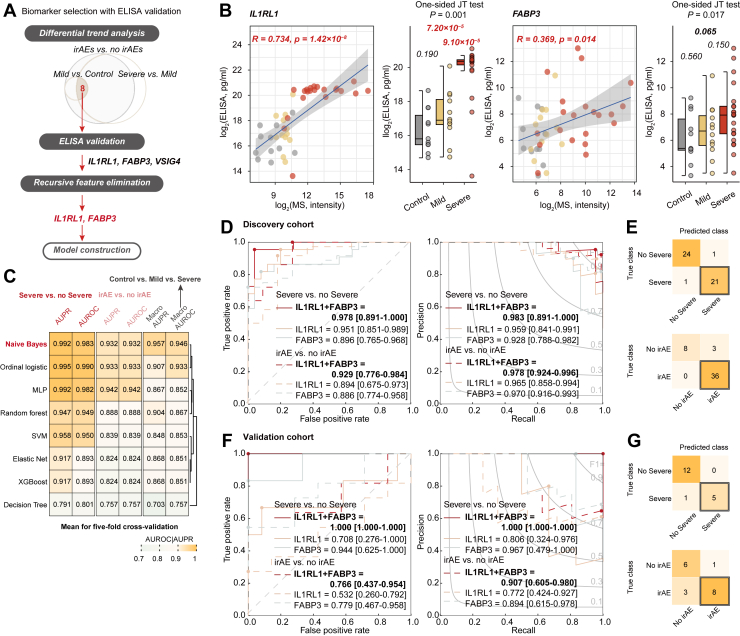
**Plasma protein biomarkers identification and model development for irAEs.***A*, Workflow for biomarker selection and validation. *B*, ELISA-validated protein abundance of IL1RL1 and FABP3 across patient samples with Pearson’s correlation in the discovery cohort. Statistical significance was evaluated using two-sided Wilcoxon rank-sum tests for group comparisons and one-sided Jonckheere-Terpstra tests for severity trend analysis. *C*, performance comparison of eight machine learning models using five-fold cross-validation. *D* and *F*, receiver–operating characteristic (ROC) and precision-recall (PR) curves of the ProIRAE modelin the discovery (*D*) and validation (*F*) cohorts. For e. g., Confusion matrix displaying classification performance of the ProIRAE model based on predicted risk scores in the discovery (*E*) and validation (*G*) cohorts.

To assess the clinical applicability and reproducibility of the eight candidate protein biomarkers identified, we quantified their plasma abundance levels in the discovery cohort using ELISA (Supplemental Table S6 and Supplemental Fig. S6*A*). Among these candidates, interleukin-1 receptor-like 1 (*IL1RL1*, protein in M1), and fatty acid-binding protein 3 (*FABP3*, protein in M16), were successfully validated by ELISA and consistently retained through recursive feature elimination. Both proteins showed significant positive correlation between ELISA and MS measurements (*IL1RL1*, R = 0.734, *p* = 1.42 × 10^-8^; *FABP3*, R = 0.369, *p* = 0.014), and demonstrated a consistent increasing abundance trend with escalating irAE severity (*IL1RL1, p* = 0.001; *FABP3, p* = 0.017) (Fig. 4, *A* and *B*).

Subsequently, we constructed eight machine learning models using *IL1RL1* and *FABP3*. Among these, the Naive Bayes classifier demonstrated superior performance in five-fold cross-validation and was selected as the final model, designated as ProIRAE (Fig. 4*C*). For the discovery cohort, the ProIRAE model demonstrated strong predictive performance, achieving an AUROC of 0.929 and an AUPR of 0.978 for distinguishing any irAEs from no irAEs, and an AUROC of 0.978 and an AUPR of 0.983 for discriminating severe irAEs from non-severe cases (Fig. 4*D*). ProIRAE accurately identified severe irAEs with a sensitivity of 95.5% (21/22) and specificity of 96.0% (24/25) in the discovery cohort (Fig. 4*E*). Similarly, for predicting any irAEs in the discovery cohort, the model achieved a sensitivity of 100.0% (36/36) and specificity of 72.0% (8/11) (Fig. 4*E* and Supplemental Table S7). The model predictions show no significant associations with key clinical variables, including age, BMI, treatment duration, sex, or disease stage (Supplemental Fig. S6*B*).

To assess generalizability, the ProIRAE was applied to an independent cohort of 18 patients. The ProIRAE maintained robust predictive performance, with an AUROC of 0.766 and an AUPR of 0.907 for distinguishing irAEs from non-irAE cases, and an AUROC of 1.000 and an AUPR of 1.000 for predicting severe irAEs (Fig. 4*F*). ProIRAE achieved a sensitivity of 83.3% (5/6) and specificity of 100.0% (12/12) for severe irAEs prediction, and a sensitivity of 72.7% (8/11) and specificity of 85.7% (6/7) for any irAEs prediction (Fig. 4*G* and Supplemental Table S8). Additionally, the combination of *IL1RL1* and *FABP3* in the ProIRAE model outperformed the predictive power of either protein biomarker alone (Fig. 4, *D* and *F*), highlighting the synergistic effect of these two protein biomarkers in predicting irAE risk and severity.

## Discussion

The broad spectrum of irAEs, coupled with their unpredictable nature and potential progression to life-threatening conditions requiring ICU admission, pose significant challenges to clinicians in detecting, monitoring, and managing irAEs (7, 14, 29). In this study, we performed a comprehensive mapping of the plasma proteomic profile of irAEs in patients receiving anti-PD-1/PD-L1 therapies, with the aim of investigating the proteomic changes associated with irAEs and identifying non-invasive biomarkers for risk prediction and stratification of irAEs, particularly those progressing to ICU-level severity.

ICIs targeting PD-1/PD-L1 have been widely used in clinical settings, and ICI-induced irAEs have been shown to share underlying molecular mechanisms and non-organ-specific biomarkers that correlate with severity (3, 18, 22, 30, 31, 32). It is noteworthy that several previously reported irAE biomarkers remained differentially expressed in plasma. These include *CRP*, which is associated with systemic inflammatory response (33); *CD177*, implicated in immune-mediated intestinal disease (34); and *LCP1,* as part of a predictor in a multi-omics research (35). Although initial efforts have been made to investigate predictive biomarkers for irAEs, previous studies have often focused on specific cancer types or immune signaling pathways (8). The loss of significance of other previously proposed biomarkers may be attributable to cohort heterogeneity or differences in detection technologies (21). Using a pan-cancer scope and label-free mass spectrometry-based quantitative proteomics technology, our study offers a more comprehensive proteomic analysis and provides valuable insights into the common molecular mechanisms underlying irAEs, increasing the generalizability of the findings.

Through network correlation analysis, we observed a consistent shift from humoral (antibody-mediated) to cellular (T cell-mediated) immunity as irAE severity increased. This shift was reflected in the identification of specific protein modules that were either upregulated or downregulated in response to irAE severity. Interestingly, we identified heterogeneous (M0) and unstable (M2) modules that showed no clear clustering across irAE groups or exhibited high within-group variability. These modules were enriched in antigen presentation pathways and immune activation, supporting the hypothesis that antigen cross-presentation between normal and immune cells may be a key mechanism underlying the considerable clinical heterogeneity of irAEs and their diverse manifestations (3, 36). Beyond the unstable proteomic modules, the M1 module, which represents a robust signature of increased immune activation and inflammatory response, is consistent with previous findings linking irAEs to inflammatory cytokine responses (27, 37, 38, 39). In contrast, the M4 module, which is associated with reduced protein abundance secreted from the liver and and declined functional in the complement and coagulation processes (40, 41). Persistent complement dysregulation may represent a novel potential diagnostic avenue that has often been overlooked in traditional studies (42), suggesting the need for further investigation into its functional relevance in ICI-induced toxicities.

We also identified two noninvasive biomarkers, *IL1RL1* and *FABP3*, which demonstrated consistent performance in both MS and ELISA validations. Although there are currently no drugs that directly target *IL1RL1*, it functions as a key receptor for interleukin-33 (*IL-33*). *IL-33* binding to *IL1RL1/ST2*, modulated with interleukin-13 (*IL-13*) in the production of transmembrane and soluble forms, activates downstream *NF-κB* and *MAPK* signaling pathways, which are central to T helper 2 cell-mediated immunity and inflammatory responses (43, 44). Moreover, soluble *ST2* (*sST2*) has emerged as a novel clinically cardiac biomarker with elevated levels associated with worse outcomes in heart failure and myocardial infarction (45), a similar underscored pattern in the context of ICI therapy-associated myocarditis (46, 47). Additionally, *FABP3* are thought to play a role in the intracellular transport of long-chain fatty acids and their acyl-CoA esters, shows differential expression in macrophages, plays a significant role in ICI-induced pneumonitis (48) and is involved in lipid droplet biogenesis and immune modulation within the tumor microenvironment (49). According to the DrugBank database, multiple drugs targeting *IL-13* and *IL-33*, or carried by *FABP3* to the heart, have been developed and either approved or are under investigation for conditions such as atopic dermatitis, asthma, chronic obstructive pulmonary disease and bacterial growth. Notably, these conditions are also manifest as irAEs following ICI therapy (5, 50, 51). This suggests that therapeutic strategies targeting the *IL1RL1* and *FABP3* may represent promising prioritized interventions for the management of certain irAEs (52), highlighting their potential role in indicating tissue damage.

Finally, the ProIRAE model we developed provides a highly effective tool for predicting the risk. The strong predictive performance of the model demonstrates its clinical utility in the identification of patients at risk for the development of irAEs of varying severities. The validation of this model in an independent cohort further supports its robustness and generalizability, reinforcing its potential as a non-invasive, high-throughput tool for early risk detection and stratification of irAEs, which is a significant limitation of current clinical practice.

Despite these promising results, our study had several important limitations. First, the inherent heterogeneity of the data, stemming from differences in cancer types, treatment regimens, and irAE categories, may introduce confounding factors that affect the consistency and interpretation of the biomarker signatures. Although the proteomic approach provides high sensitivity and broad coverage of the plasma proteome, this heterogeneity, along with technical challenges, complicates the identification of certain robust irAE biomarkers, particularly those associated with autoimmune responses. Second, despite the pan-cancer perspective of our study, the relatively small sample size and single-center design may introduce potential biases, limiting the generalizability of our findings to broader patient populations. Third, while our design was optimized to identify diagnostic and severity-stratification biomarkers, further studies are still needed for systematic pre-treatment and serial longitudinal samples to define the predictive value of these proteomic changes and to model the dynamic evolution of the plasma proteome leading to irAE onset. Although an independent validation cohort confirmed the utility of the ProIRRAE model, further validation in larger, multi-center cohorts incorporating longitudinal samples and pre-treatment baselines is necessary to refine and establish its clinical applicability across diverse settings and cancer types.

## Conclusion

Our study provides a comprehensive plasma proteomic landscape of ICI-related adverse events and identifies validated biomarkers with clinical potential for risk stratification and management of irAEs. The development of ProIRAE, a robust noninvasive risk prediction tool, holds great promise for improving the clinical safety management of immunotherapy by enabling the effective monitoring and risk stratification of irAEs, particularly those progressing to ICU severity.

## Data Availability

Proteomic data are available from the OMIX, China National Center for Bioinformation/Beijing Institute of Genomics, Chinese Academy of Sciences (53, 54) (https://ngdc.cncb.ac.cn/omix: accession no. OMIX011761). The code for statistical analysis, modeling, and visualization presented in this manuscript and the corresponding figure panels and tables are publicly available on GitHub at https://github.com/ZhoulabCPH/ProIRAE.

## Supplemental Data

This article contains supplemental data.

## Conflict of Interest

The authors declare that they do not have any conflicts of interest with the content of this article.
