# Supplementary material for: Plasma Proteome-Driven Liquid Biopsy for Individualized Monitoring and Risk Stratification of Immune-Related Adverse Events in Checkpoint Immunotherapy
Source: Mol Cell Proteomics. 2025 Dec 13;25(1):101488. doi: 10.1016/j.mcpro.2025.101488 (PMC12818211; doi:10.1016/j.mcpro.2025.101488)
Supplement: Supplementary Information [file mmc10.docx]

Supplementary Information

**Supplementary Figures 1 to 6**

1. Supplementary Figure 1. Correlation analysis of clinical features and proteomic profiling of participants receiving ICI therapies.
2. Supplementary Figure 2. Enriched GO terms of proteins with differential abundance trends across irAE groups.
3. Supplementary Figure 3. The consensus protein co-expression network analysis.
4. Supplementary Figure 4. GO analysis for hypergeometric test-identified proteins in irAE-related network module.
5. Supplementary Figure 5. Associated proteins in M1/M4-specific KEGG and Reactome terms displayed in Figure 3c.
6. Supplementary Figure 6. Protein abundance of other candidate biomarkers detected by ELISA in plasma samples and clinical heterogeneity for predicted risk of irAE.

**Summary of Supplementary Tables 1 to 8 with details in https://github.com/ZhoulabCPH/ProIRAE_SI_**

1. Supplementary Table 1. Organ-specific immune-related adverse events identified according to the National Comprehensive Cancer Network Guidelines Version 1.2020.
2. Supplementary Table 2. The results of differential trendency proteins analysis.
3. Supplementary Table 3. Ontology, KEGG, and Reactome enrichment analyses for detected irAE-related differential trendency proteins.
4. Supplementary Table 4. The module class generated from WGCNA analysis
5. Supplementary Table 5. Ontology, KEGG, and Reactome enrichment analyses for irAE-related proteins with strong trends co-detected in WGCNA modules
6. Supplementary Table 6. ELISA validation for discovery cohort (n = 47).
7. Supplementary Table 7. Predicted values for discovery cohort.
8. Supplementary Table 8. Predicted values for validation cohort.

**
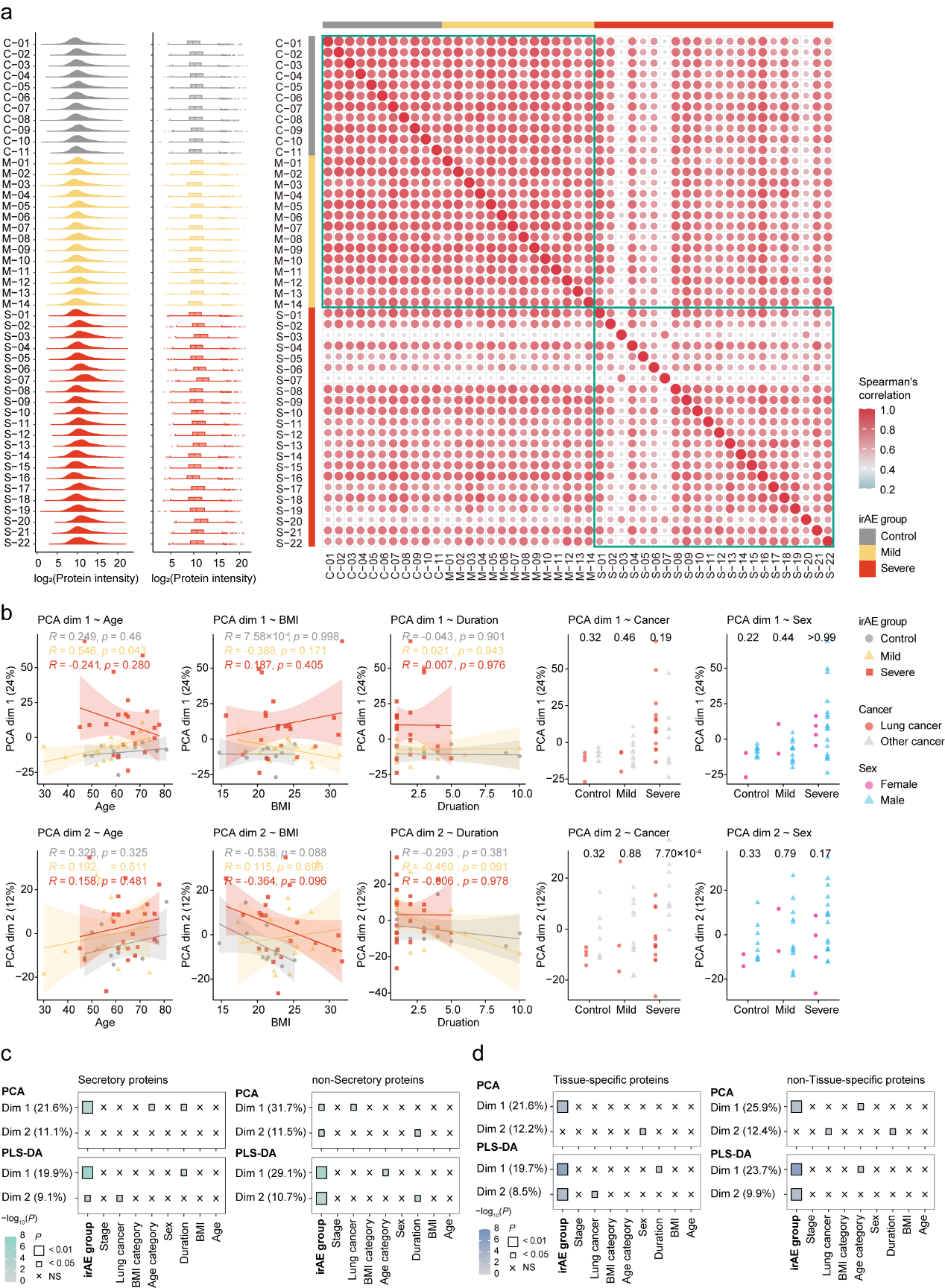
**

**Supplementary Figure 1. Correlation analysis of clinical features and proteomic profiling of participants receiving ICI therapies. a.** Proteomic profiling correlations across participants. **b.** Correlation analysis of clinical features and proteomic profiling of patients across different irAE severity groups. **c, d.** Correlation analysis of clinical features and comparable secretory and tissue-specific proteomic profiling. The proteomic profiling was evaluated with PCA/PLS-DA scores. Statistical significance assessed using Pearson correlation coefficients for continuous variables, Fisher's exact test for categorical variables, and Wilcoxon or Kruskal-Wallis tests for continuous versus categorical variables.

**
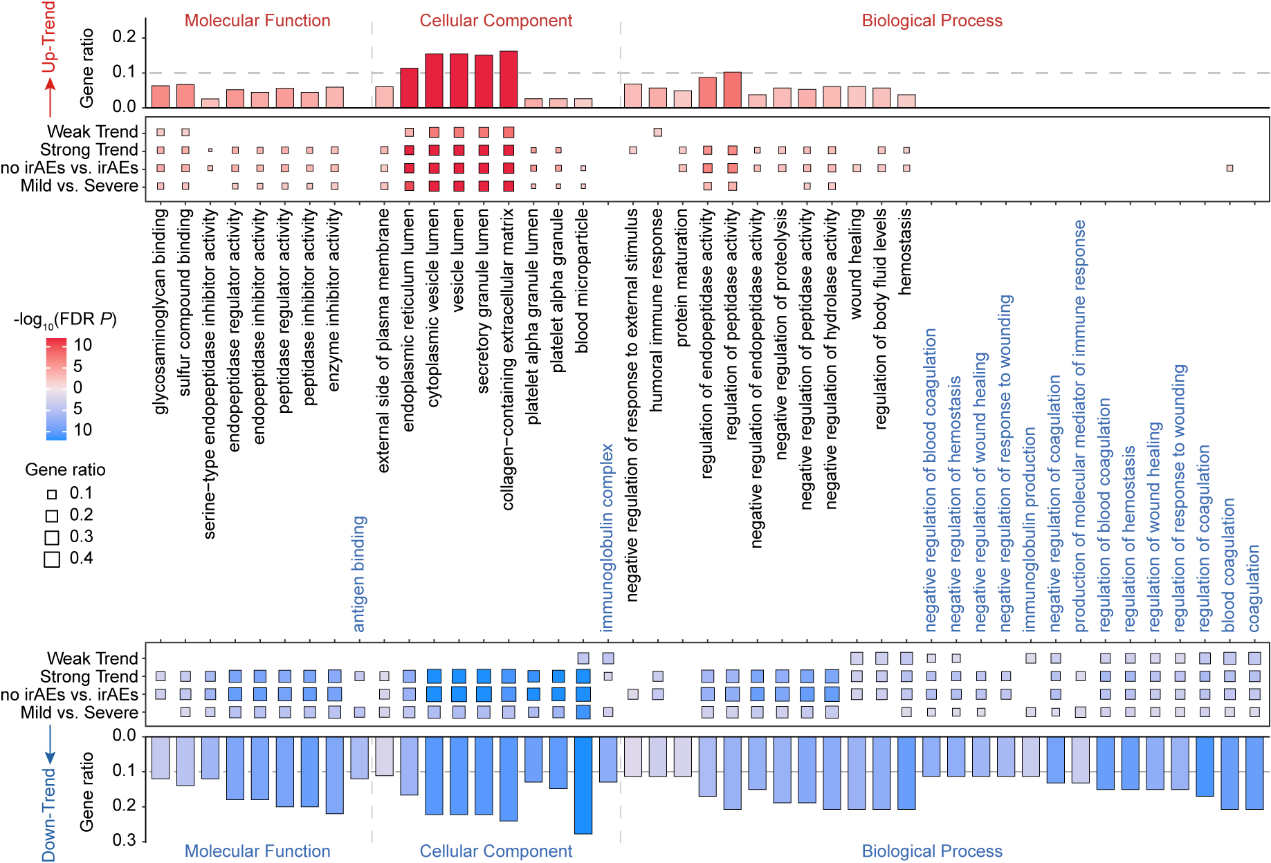
**

**Supplementary Figure 2. Enriched GO terms of proteins with differential abundance trends across irAE groups.** Terms are marked as decreased (blue) or increased (red) (FDR *P* < 0.05 and gene ratio > 0.1).


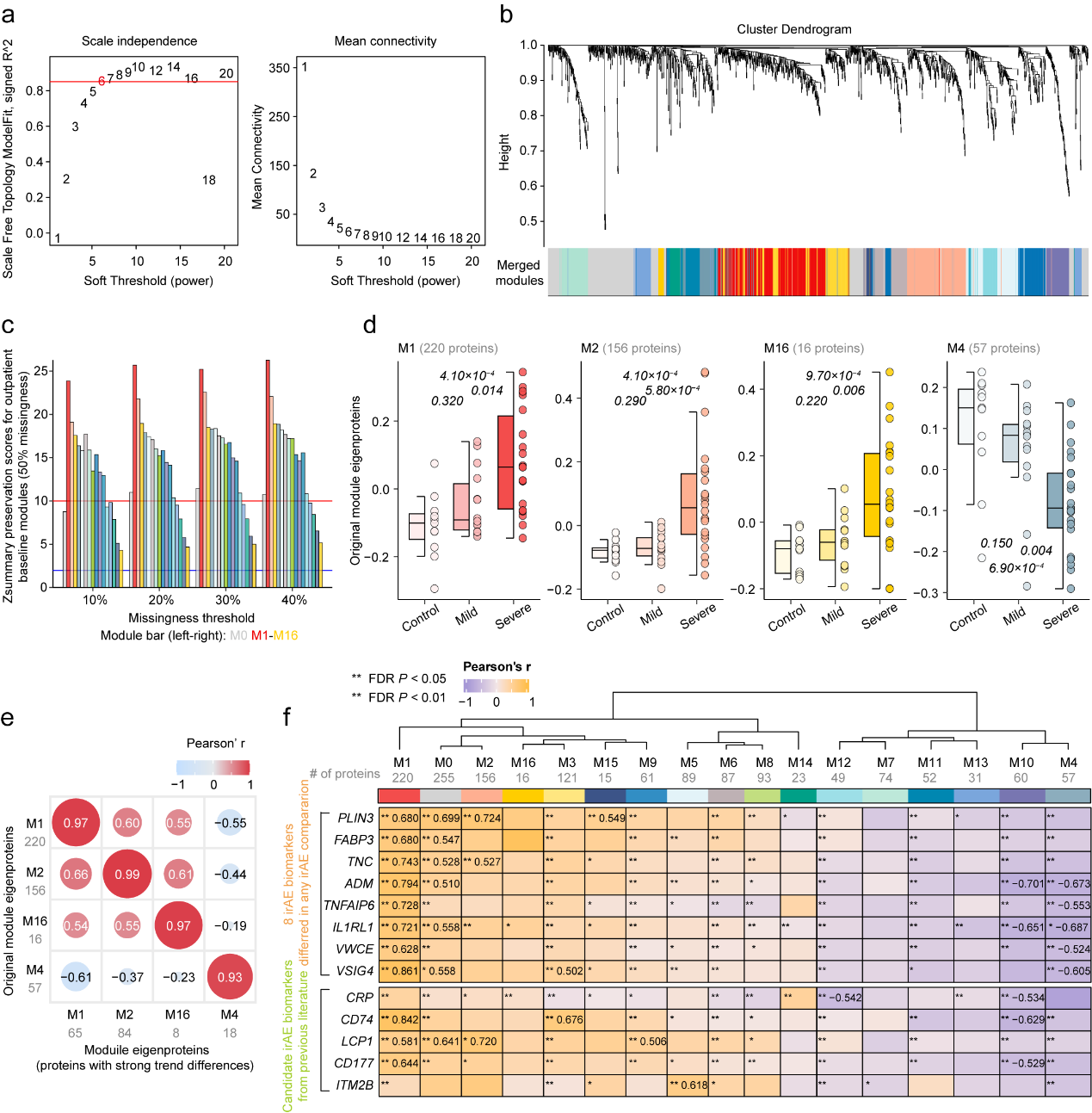


**Supplementary Figure 3. The consensus protein co-expression network analysis. a.** Determination of soft-threshold power in the consensus protein co-expression network analysis. Analysis of the scale-free index for various soft-threshold powers (β) and the mean connectivity for various soft-threshold powers. **b.** Dendrogram of proteins based on the measurement of dissimilarity and identification of the 17 modules. **c.** The effect of missing value threshold (10-40% missingness) on irAE network modules, assessed by Zsummary score. The dashed blue (1.96) and red (10) lines indicates a weakly and highly statistically significant, respectively. **d.** Box plots displayed the differences of original module eigenprotein (the first principal components of module protein expression) across irAE groups in irAE-related modules, identified in Figure. 3a. **e.** Correlation analysis of (module explanation) original module eigenproteins and those module eigenproteins generated from hypergeometric test-selected proteins. **f.** Correlation analysis of potential biomarkers and the consensus network module eigenproteins.


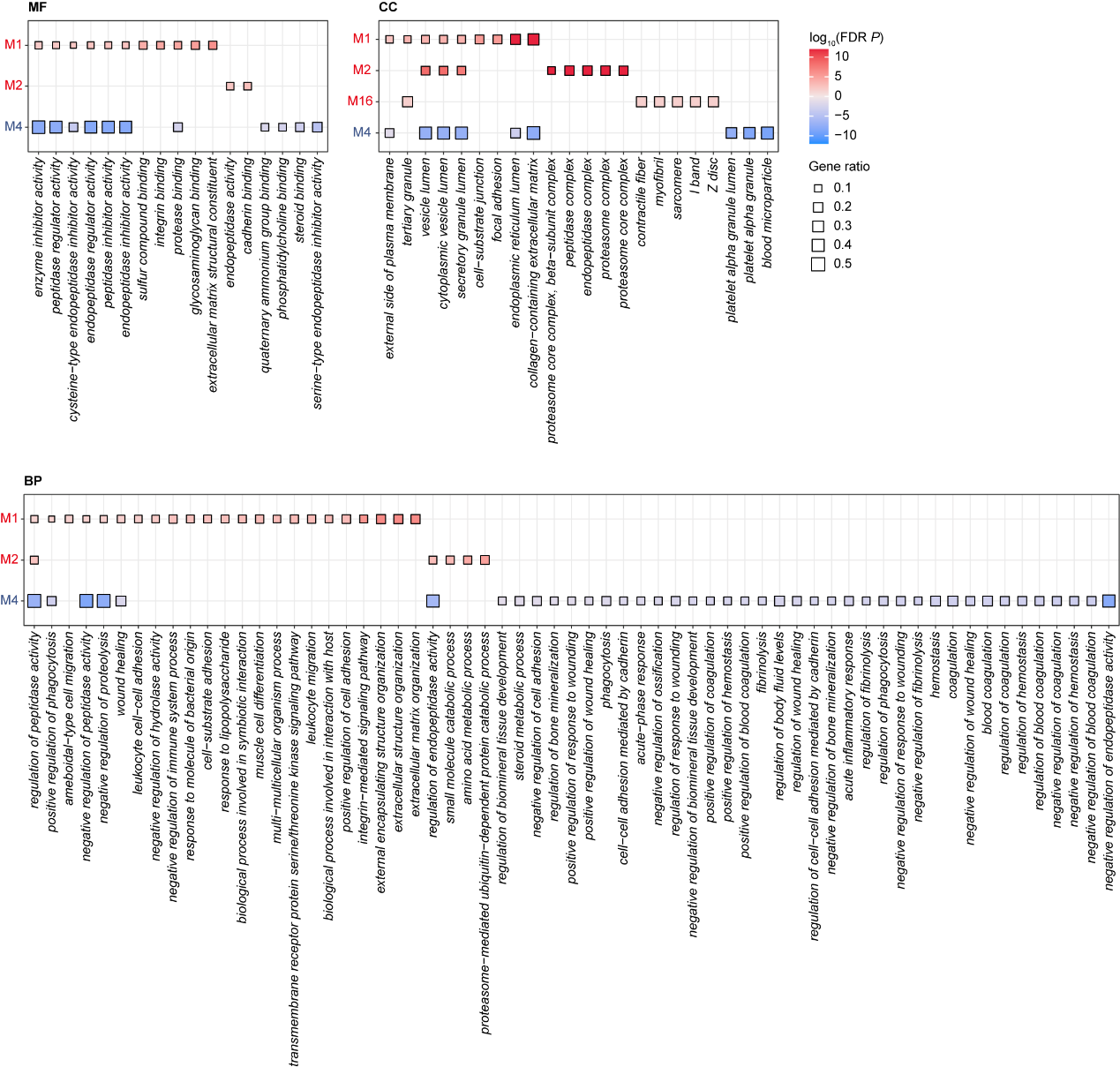


**Supplementary Figure 4. GO analysis for hypergeometric test-identified proteins in irAE-related network module.** Terms are marked as decreased (blue) or increased (red) (FDR *P* < 0.05 and gene ratio > 0.1). The detail results of enrichment analysis were presented in Supplementary Table. 5.

**
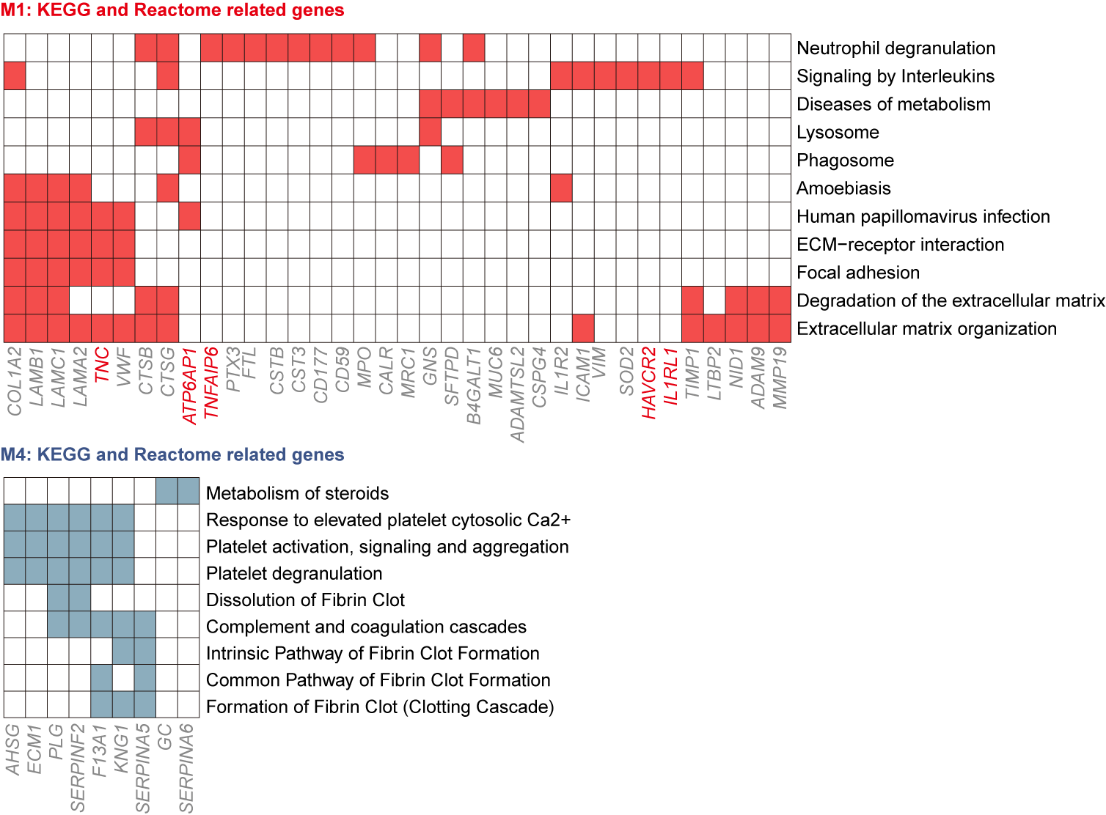
**

**Supplementary Figure 5. Associated proteins in M1/M4-specific KEGG and Reactome terms displayed in Figure 3c.** Colored proteins were those significantly change in Control vs. Mild, indicating the potential ability on early detection for patients suffering from irAEs.


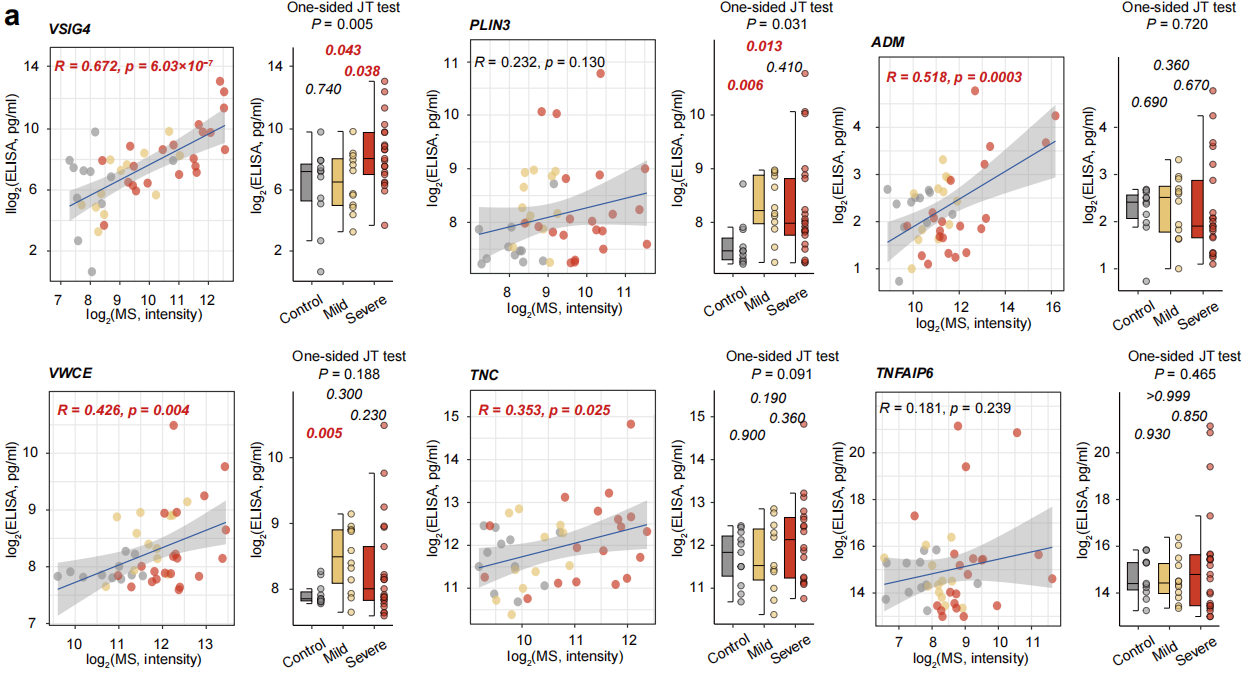


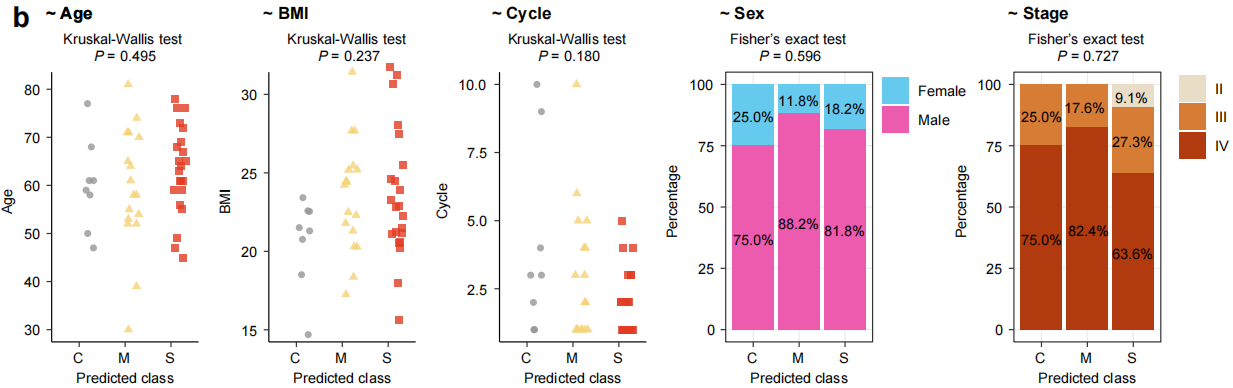


**Supplementary Figure 6. Protein abundance of other candidate biomarkers detected by ELISA in plasma samples and clinical heterogeneity for predicted risk of irAE.** Statistical significance was assessed using two-sided Wilcoxon tests and one-sided Jonckheere-Terpstra tests. Based on our established selection criteria, PLIN3 and TNFAIP were excluded due to a lack of concordance between MS and ELISA in paired samples (without significant correlation). Furthermore, ADM, VWCE, and TNC were removed as their abundance patterns were inconsistent with the expected upward trend associated with increasing irAE severity.
